# Lymphopenia in severe coronavirus disease-2019 (COVID-19): systematic review and meta-analysis

**DOI:** 10.1186/s40560-020-00453-4

**Published:** 2020-05-24

**Authors:** Ian Huang, Raymond Pranata

**Affiliations:** 1 Faculty of Medicine, Universitas Pelita Harapan, Tangerang, Banten Indonesia; 2 Department of Internal Medicine, Hasan Sadikin General Hospital-Faculty of Medicine, Universitas Padjadjaran, Bandung, Indonesia

**Keywords:** Coronavirus, COVID-19, Lymphocyte count, Lymphopenia, SARS-CoV-2

## Abstract

**Objective:**

Clinical and laboratory biomarkers to predict the severity of coronavirus disease 2019 (COVID-19) are essential in this pandemic situation of which resource allocation must be urgently prepared especially in the context of respiratory support readiness. Lymphocyte count has been a marker of interest since the first COVID-19 publication. We conducted a systematic review and meta-analysis in order to investigate the association of lymphocyte count on admission and the severity of COVID-19. We would also like to analyze whether patient characteristics such as age and comorbidities affect the relationship between lymphocyte count and COVID-19.

**Methods:**

Comprehensive and systematic literature search was performed from PubMed, SCOPUS, EuropePMC, ProQuest, Cochrane Central Databases, and Google Scholar. Research articles in adult patients diagnosed with COVID-19 with information on lymphocyte count and several outcomes of interest, including mortality, acute respiratory distress syndrome (ARDS), intensive care unit (ICU) care, and severe COVID-19, were included in the analysis. Inverse variance method was used to obtain mean differences and its standard deviations. Maentel-Haenszel formula was used to calculate dichotomous variables to obtain odds ratios (ORs) along with its 95% confidence intervals. Random-effect models were used for meta-analysis regardless of heterogeneity. Restricted-maximum likelihood random-effects meta-regression was performed for age, gender, cardiac comorbidity, hypertension, diabetes mellitus, COPD, and smoking.

**Results:**

There were a total of 3099 patients from 24 studies. Meta-analysis showed that patients with poor outcome have a lower lymphocyte count (mean difference − 361.06 μL [− 439.18, − 282.95], *p* < 0.001; *I*^2^ 84%) compared to those with good outcome. Subgroup analysis showed lower lymphocyte count in patients who died (mean difference − 395.35 μL [− 165.64, − 625.07], *p* < 0.001; *I*^2^ 87%), experienced ARDS (mean difference − 377.56 μL [− 271.89, − 483.22], *p* < 0.001; *I*^2^ 0%), received ICU care (mean difference − 376.53 μL [− 682.84, − 70.22], *p* = 0.02; *I*^2^ 89%), and have severe COVID-19 (mean difference − 353.34 μL [− 250.94, − 455.73], *p* < 0.001; *I*^2^ 85%). Lymphopenia was associated with severe COVID-19 (OR 3.70 [2.44, 5.63], *p* < 0.001; *I*^2^ 40%). Meta-regression showed that the association between lymphocyte count and composite poor outcome was affected by age (*p* = 0.034).

**Conclusion:**

This meta-analysis showed that lymphopenia on admission was associated with poor outcome in patients with COVID-19.

## Introduction

Coronavirus disease 2019 (COVID-19) has been declared by the World Health Organization (WHO) as a global public health emergency due to its pandemicity [1]. Since its first emergence in Wuhan, China, more than 450,000 cases and 20,000 deaths have been recorded globally due to COVID-19 [2]. While most patients with COVID-19 have mild influenza-like illness and may be asymptomatic, a minority of patients will develop severe pneumonia, acute respiratory distress syndrome (ARDS), multi-organ failure (MOF), and death [3]. Clinical and laboratory biomarkers [4] to predict the mortality and severity of COVID-19 are essential in this pandemic situation of which resource allocation must be urgently prepared especially in the context of respiratory support readiness.

Since the first descriptive study in China regarding the COVID-19 infection [5], lymphocyte count has been a marker of interest. It has been associated with severe COVID-19 [6, 7], and non-survivors of COVID-19 were reported to have a significantly lower lymphocyte count than survivors [7]. Whether lower lymphocyte count and lymphopenia could really be predictor of severity of COVID-19 was our main interest, since this laboratory tools are readily available even in the remote areas. Therefore, in the present study, we conducted a systematic review and meta-analysis in order to investigate the association of lymphocyte count on admission and the severity of COVID-19. We would also like to analyze whether patient characteristics such as age and comorbidities affect the relationship between lymphocyte count and COVID-19.

## Material and methods

### Eligibility criteria

We included research articles concerning adult patients diagnosed with COVID-19 that has information on lymphocyte count at admission, and clinical grouping or outcome of clinically validated definition of severe COVID-19, death, or ICU care. We exclude review articles, non-research letters, commentaries, case reports, animal studies, original research with samples below 20 or case reports and series, non-English language articles, and studies in pediatric populations (≤ 17 years old).

### Search strategy and study selection

We systematically searched PubMed, SCOPUS, EuropePMC, ProQuest, Cochrane Central Databases, and Google Scholar with the search terms “COVID-19” OR “SARS-CoV-2” AND “Lymphocyte” (Table S1). After initial search, duplicates were excluded. Two independent authors (IH and RP) screened title and abstracts for potentially relevant articles. The full-text of the potential articles was assessed by applying inclusion and exclusion criteria. The literature search was finalized on March 25, 2020. The study was carried out in accordance with the declaration of Helsinki and with the term of local protocol. This is a Preferred Reporting Items for Systematic Reviews and Meta-Analyses (PRISMA)-compliant systematic review and meta-analysis

### Data extraction

Data extraction was performed independently by two authors (IH and RP). We used standardized forms that included author, year, study design, age, gender, cardiac comorbidities, hypertension, diabetes mellitus, chronic obstructive pulmonary disease, smoking, lymphocyte count, lymphopenia, mortality, ARDS, ICU care, and severe COVID-19.

The outcome of interest was composite poor outcome that comprised of mortality, ARDS, ICU care, and severe COVID-19. Mortality and ICU care was defined as death and admittance to ICU during inhospital care, respectively. ARDS was defined according to the criteria from the World Health Organization (WHO) interim guidance for severe acute respiratory infection (SARI) in COVID-19, which includes the acuity of symptom onset, chest X-ray and origin of pulmonary infiltrates, and oxygenation impairment [8]. Severe COVID-19 was defined as patients who had any of the following features at the time of, or after, admission: (1) respiratory rate ≥ 30 breaths per min, (2) oxygen saturation ≤ 93% (at rest), (3) ratio of partial pressure of arterial oxygen to fractional concentration of oxygen inspired air (PaO2 to fiO2 ratio) ≤ 300 mmHg, or (4) specific complications, such as septic shock, respiratory failure, and or multiple organ dysfunction [9].

### Statistical analysis

The meta-analysis of studies was performed using Review Manager 5.3 (Cochrane Collaboration) and Stata version 16. To pool continuous variables, we used an inverse variance method to obtain mean differences (MDs) and its standard deviations (SDs). Maentel-Haenszel formula was used to calculate dichotomous variables to obtain odds ratios (ORs) along with its 95% confidence intervals (CIs). We used random-effects models for pooled analysis regardless of heterogeneity. All *P* values were two-tailed, and statistical significance was set at ≤ 0.05. Subgroup analysis was performed for lymphopenia cutoff point at ≤ 1100 cells/μL. Sensitivity analysis using a leave-one-out method was performed to single out the cause of heterogeneity. Regression-based Egger’s test was used to assess small-study effects for continuous variables and Harbord’s test for binary outcome. Restricted maximum likelihood random-effects meta-regression was performed for age, gender, cardiac comorbidity, hypertension, diabetes mellitus, chronic obstructive pulmonary disease (COPD), and smoking.

## Results

### Baseline characteristics and study selection

**Fig. 1:**
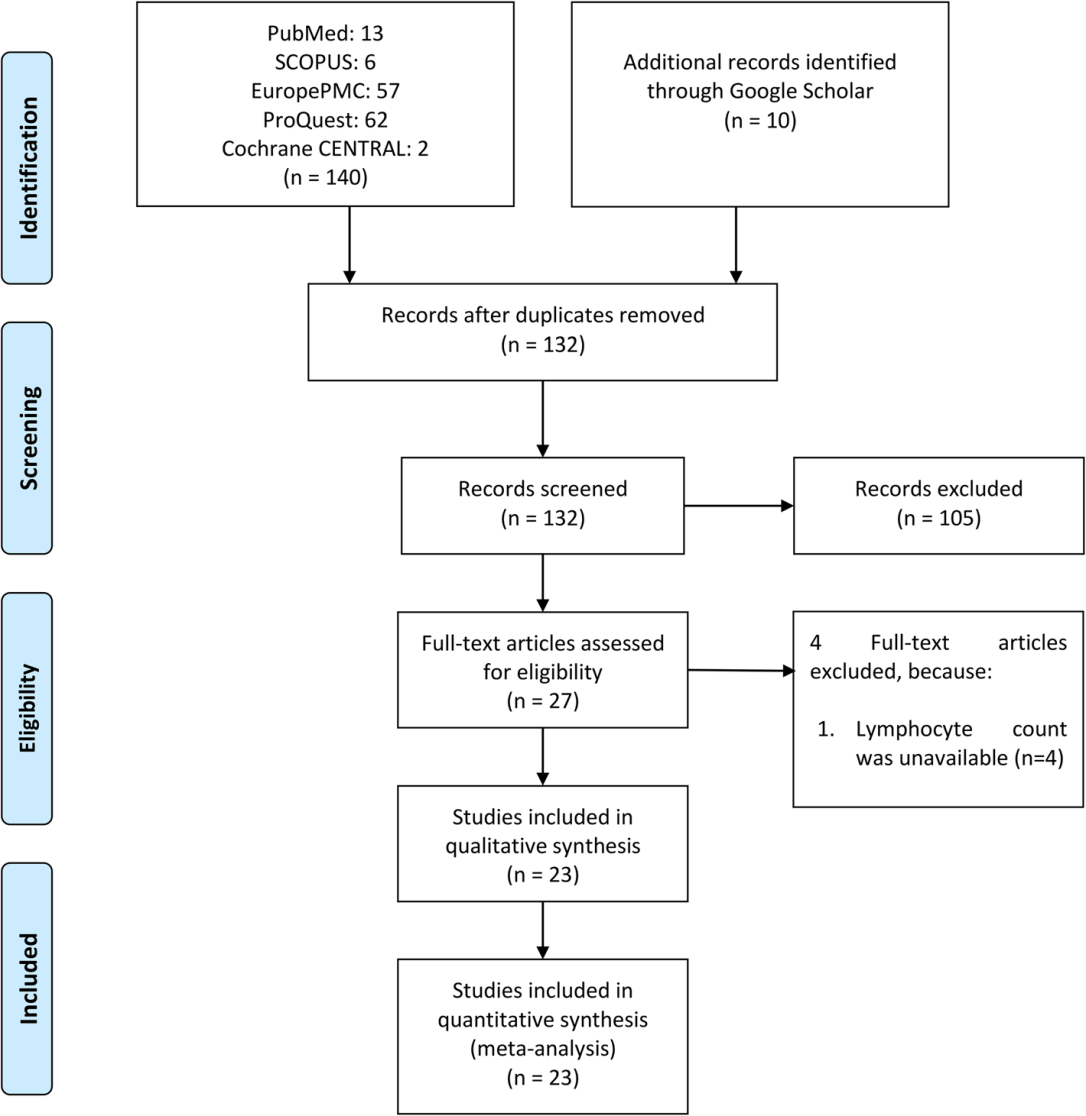
Study flow diagram

We found a total of 150 records of which 132 remained after the removal of duplicates. A total of 105 records were excluded after screening the title/abstracts. After assessing 27 articles for eligibility, we excluded 4 in which lymphocyte count was unavailable. Thereby, 23 studies remained for qualitative synthesis and meta-analysis (Fig. 1). There were a total of 3099 patients from 23 studies [5–7, 10–29]. Baseline characteristics are presented in Table 1. The reported mean age of the patients on these studies was 51 years old; 55% of the overall samples were men. Most studies reported lymphocyte count on admission except for Ruan et al. and Liu et al. whom did not state the period of blood test. Nevertheless, their study presumably reported lymphocyte counts on admission.

**Table 1:** Baseline characteristics of the included studies

Author	Study design	Country	Sample (n)	Gender (male %)	Age (years)	Cardiac comorbidity (%)	HT (%)	DM (%)
Ruan et al. 2020 [7]	Retrospective	Wuhan, China	150	102/150 (68%)	67 non-survivor and 50 survivor (median)	8.7	34.7	16.7
Yang et al. 2020 [6]	Retrospective	Wuhan, China	52	35/52 (67%)	59.7 (mean)	9.6	NR	17.3
Zhou et al. 2020 [20]	Retrospective	Wuhan, China	191	119/191 (62%)	56 (median)	7.9	30.4	18.8
Chen et al. 2020 [23]	Retrospective	Wuhan, China	124	61/124 (49%)	72 non-survivor and 53 survivor (median)	16.1	33.1	11.3
Huang et al. 2020 [5]	Retrospective	Wuhan, China	41	30/41 (73%)	49 (median)	14.6	14.6	19.5
Wang et al. 2020 [24]	Retrospective	Wuhan, China	138	75/138 (54%)	56 (median)	14.5	31.2	10.1
Cao et al. 2020 [25]	Retrospective	Shanghai, China	198	101/198 (51%)	50.1 (mean)	6.1	21.2	7.6
Wu et al. 2020 [26]	Retrospective	Wuhan, China	201	30/41 (73%)	51 (median)	4.0	19.4	10.9
Yanli et al. 2020 [27]	Retrospective	Wuhan, China	109	59/109 (54%)	55 (median)	6.4	33.9	11.0
Guan et al. 2020 [28]	Retrospective	Wuhan, China	1099	640/1099 (58%)	47 (median)	2.5	15.0	7.4
Liu et al. 2020 [29]	Retrospective	Wuhan, China	78	39 (58%)	38 (median)	NR	10.3	6.4
Zhang G et al 2020 [10]	Retrospective	Wuhan, China	221	108/221 (48.9%)	55 (median)	10.0	24.4	10.0
Zhang et al. 2020 [11]	Retrospective	Wuhan, China	140	71/140 (50.7%)	57 (median)	5.0	30.0	12.1
Wan et al. 2020 [12]	Retrospective	Chongqing, China	135	72/135 (53.3%)	47 (median)	5.0	9.6	8.9
Qu et al. 2020 [13]	Retrospective	Huizhoi, China	30	16/30 (53.3%)	50.5 (median)	NR	NR	NR
Qin et al. 2020 [14]	Retrospective	Wuhan, China	452	235/ 452 (52%)	58 (median)	6.0	29.9	16.6
Wang et al. 2020 [15]	Retrospective	Wuhan, China	110	48/110 (43%)	≤ 40 (53%), 41–60 (21%), > 60 (36%)	6.4	20.9	13.6
Feng et al. 2020 [16]	Retrospective	Wuhan, China	141	72/141 (51.1%)	44 (median)	2.7	14.9	5.7
Lei et al. 2020 [17]	Retrospective	Chongqing, China	51	32/51 (62.7%)	45 (median)	NR	7.8	7.8
Liu et al. 2020 [18]	Retrospective	Wuhan, China	40	15/40 (37.5%)	48.7 (mean)	NR	15.0	15.0
Cai et al. 2020 [19]	Retrospective	Shenzhen, China	298	149/298 (50%)	47 (median)	NR	NR	NR
Liu et al. 2020 [21]	Prospective	Beijing, China	61	31/61 (50.8)	40 (median)	1.6	19.7	8.2
Tabata et al. 2020 [22]	Retrospective	Tokyo, Japan	104	47/104 (45%)	68 (median)	NR	NR	NR

*NR* not reported, *DM* diabetes mellitus, *HT* hypertension

### Lymphocyte count and poor outcome

**Fig. 2:**
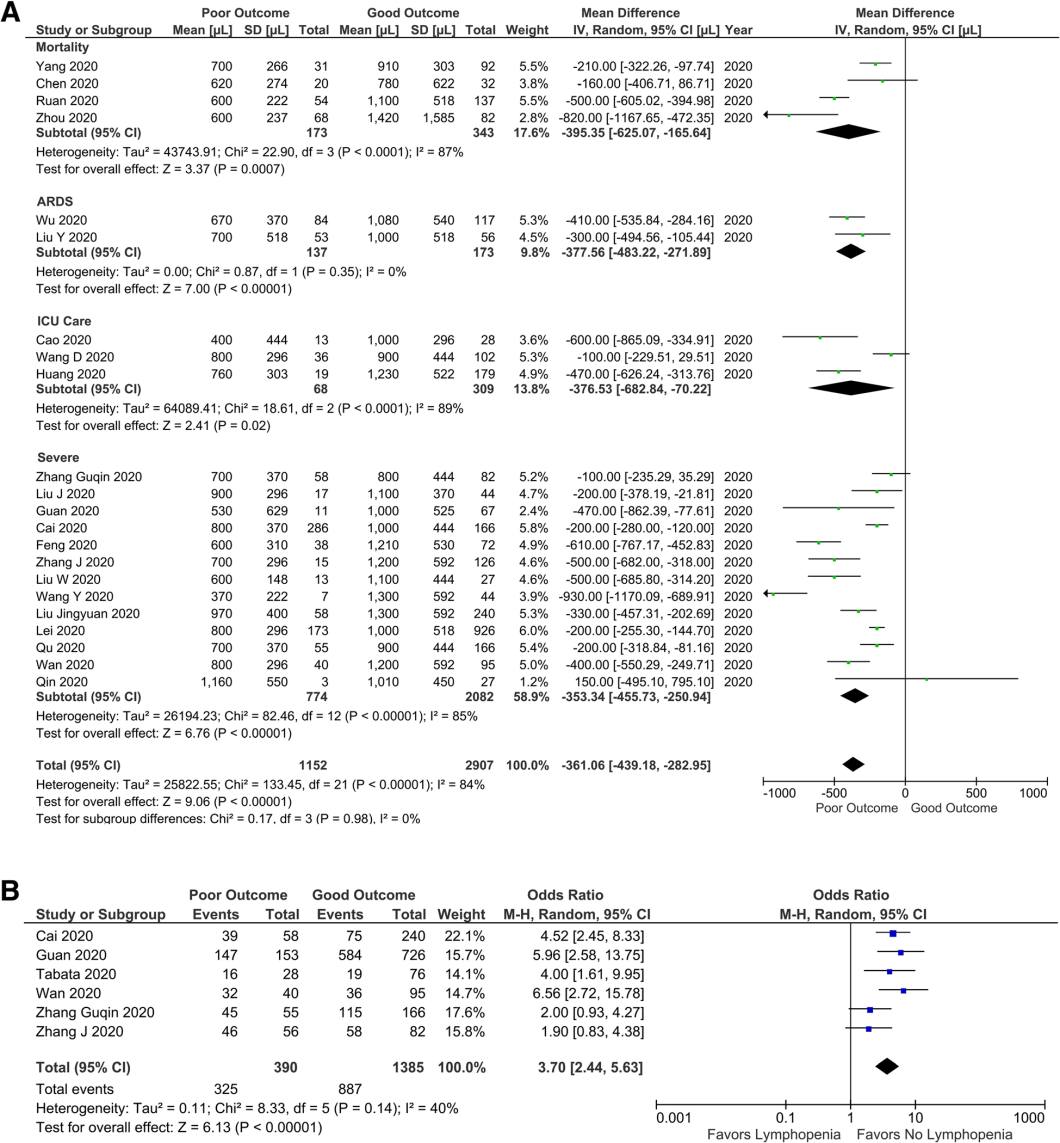
Lymphocyte count and composite poor outcome. Patients with composite poor outcome comprising of mortality, ARDS, need for ICU care, and severe COVID-19 has lower lymphocyte count. ARDS: acute respiratory distress syndrome, ICU: intensive care unit

Meta-analysis showed that patients with poor outcome had a lower lymphocyte count (mean difference − 361.06 μL [− 439.18, − 282.95], *p* < 0.001; *I*^2^ 84%, *p* < 0.001) compared to those with good outcome (Fig. 2a). Subgroup analysis showed that patients that died had lower lymphocyte count (mean difference − 395.35 μL [− 165.64, − 625.07], *p* < 0.001; *I*^2^ 87%, *p* < 0.001). Patients with ARDS had lower lymphocyte count (mean difference − 377.56 μL [− 271.89, − 483.22], *p* < 0.001; *I*^2^ 0%, *p* = 0.35). Patients in ICU care had lower lymphocyte count (mean difference − 376.53 μL [− 682.84, − 70.22], *p* = 0.02; *I*^2^ 89%, *p* < 0.001). Patients with severe COVID-19 had lower lymphocyte count compared to non-severe COVID-19 patients (mean difference − 353.34 μL [− 250.94, − 455.73], *p* < 0.001; *I*^2^ 85%, *p* < 0.001) (Table 2).

**Table 2:** Lymphocyte count and outcome of the included studies

Author	Smoking (%)	COPD (%)	Poor outcome (%)	Lymphocyte count in good outcome (/μL)	Lymphocyte count in poor outcome (/μL)	Lymphopenia cutoff	Lymphopenia in good outcome (%)	Lymphopenia in poor outcome (%)
Ruan et al. 2020 [7]	NR	2.0	68/150 (45%), death	1420 (2140)	600 (320)	NR	NR	NR
Yang et al. 2020 [6]	4	7.6 (CLD)	20/52 (38%), death	740 (840)	620 (370)	NR	NR	NR
Zhou et al. 2020 [20]	5.7	3.1	54/191 (28%), death	1100 (800–1500)	600 (500–800)	NR	NR	NR
Chen et al. 2020 [23]	13.8	4.9	31/123 (25%), death	910 (410)	700 (360)	NR	NR	NR
Huang et al. 2020 [5]	7.3	2.4	13/41 (31%), ICU	1000 (700–1100)	400 (200–800)	≤ 1000	15/28 (54%)	11/13 (85%)
Wang et al. 2020 [24]	NR	2.9	36/138 (26%), ICU	900 (600–1200)	800 (500–900)	NR	NR	NR
Cao et al. 2020 [25]	5.6	NR	19/198 (9%), ICU	1230 (860–1565)	760 (530–940)	≤ 1000	1/179 (0.6%)	16/19 (84%)
Wu et al. 2020 [26]	NR	2.5 (CLD)	84/201 (41%), ARDS	1080 (720–1450)	670 (490–990)	NR	NR	NR
Liu et al. 2020 [27]	NR	3.7	53/109 (48%), ARDS	1000 (800–1400)	700 (400–1100)	NR	NR	NR
Guan et al. 2020 [28]	14.4	1.1	173/1099 (15%), severe	1000 (800–1400)	800 (600–1000)	≤ 1500	584/726 (83.2%)	147/153 (96.1%)
Liu et al. 2020 [29]	6.4	10.0	11/78 (14%), severe	1000 (680–1390)	530 (300–1150)	NR	NR	NR
Zhang et al. 2020 [10]	NR	2.7	55/221 (24%), severe	900 (600–1200)	700 (400–900)	≤ 1100	115/166 (69%)	48/55 (87%)
Zhang et al. 2020 [11]	6.4	1.4	58/140 (34%), severe	800 (600–1200)	700 (500–1000)	≤ 1100	58/82 (70.%)	46/56 (82.1%)
Wan et al. 2020 [12]	6.7	0.7 (CLD)	40/135 (29%), severe	1200 (800–1600)	800 (600–1000)	≤ 1100	36/95 (38%)	32/40 (80%)
Qu et al. 2020 [13]	NR	NR	3/30 (10%), severe	1010 (450)	1160 (550)	NR	NR	NR
Qin et al. 2020 [14]	1.5	2.6	286/452 (63%), severe	1000 (700–1300)	800 (600–1100)	NR	NR	NR
Wang et al. 2020 [15]	23.6	5.4	38/110 (34%), severe	1210 (530)	600 (310)	NR	NR	NR
Feng et al. 2020 [16]	4.9	2.8	15/141 (10%), severe	1200 (800–1600)	700 (600–1000)	NR	NR	NR
Lei et al. 2020 [17]	NR	NR	7/51 (13%), severe	1300 (900–1700)	370 (300–600)	NR	NR	NR
Liu et al. 2020 [18]	NR	NR	13/40 (32.5%), severe	1100 (800–1400)	600 (600–800)	NR	NR	NR
Cai et al. 2020 [19]	NR	NR	58/298 (19%), severe	1300 (1000–1800)	970 (650–1190)	≤ 1100	75/240 (31.3%)	39/58 (67%)
Liu et al. 2020 [21]	6.6	8.2	17/61 (27%), severe	1100 (900–1400)	900 (700–1100)	NR	NR	NR
Tabata et al. 2020 [22]	17.3	6.7 (CLD)	28/104 (26%), severe	NR	NR	< 1200	19/76 (25%)	16/28 (57.1%)

Lymphocyte count presented as median (IQR) or mean (SD). Smoking includes current and/or former smoker

*NR* not reported, *CLD* chronic lung disease/pulmonary disease

Sensitivity analysis showed that removal of one particular study [24] reduced the heterogeneity for ICU outcome, but lymphocyte count was still lower in those that received ICU care (mean difference − 503.51 μL [− 638.11, − 368.92], *p* < 0.001; *I*^2^ 0%, *p* = 0.41). Removal of any single study did not significantly reduce heterogeneity for mortality, ARDS, and severe COVID-19.

### Lymphopenia and severe COVID-19

Meta-analysis showed that lymphopenia was associated with severe COVID-19 (OR 3.70 [2.44, 5.63], *p* < 0.001; *I*^2^ 40%, *p* = 0.14) (Fig. 2b). Subgroup analysis was performed for lymphopenia with definition of lymphocyte count ≤ 1100 μL, showing that lymphopenia was associated with severe COVID-19 (OR 3.27 [1.85, 5.78], *p* < 0.001; *I*^2^ 55%, *p* = 0.08) (Figure S1).

### Meta-regression

**Fig. 3:**
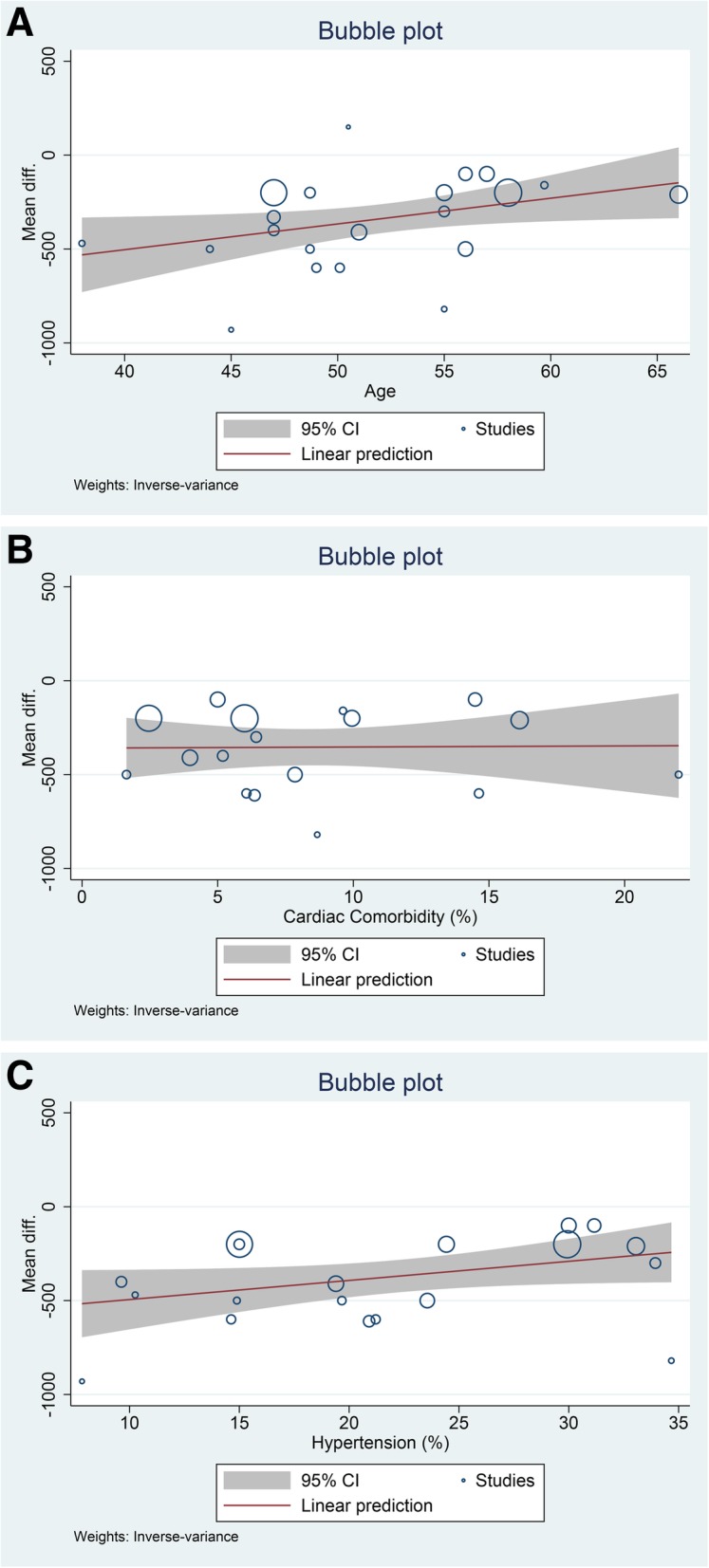
Lymphopenia and severe COVID-19. Lymphopenia was associated with severe COVID

Random-effects meta-regression analysis showed that the association between lower lymphocyte count in patients with composite poor outcome was affected by age (*p* = 0.034) (Fig. 3a), but not by gender (*p* = 0.109), cardiac comorbidity (*p* = 0.953) (Fig. 3b), hypertension (*p* = 0.065) (Fig. 3c), diabetes mellitus (*p* = 0.931), COPD (*p* = 0.798), and smoking (*p* = 0.581).

### Subgroup analysis for age

Since the composite poor outcome was affected by age, we performed subgroup analysis by using 55 years old as cutoff point. The difference in lymphocyte count in < 55 years old (mean difference − 378.93 μL [− 472.58, − 285.27], *p* < 0.001; *I*^2^ 81%, *p* < 0.001) was greater compared to those in ≥ 55 years old (mean difference − 341.68 μL [− 484.25, − 199.11], *p* < 0.001; *I*^2^ 87%, *p* < 0.001). The association between lymphopenia and severe COVID-19 was stronger in < 55 years old (OR 5.32 [3.46, 8.18], *p* < 0.001; *I*^2^ 0%, *p* = 0.75) compared to ≥ 55 years old (OR 2.38 [1.48, 3.84], *p* < 0.001; *I*^2^ 0%, *p* = 0.42).

### Publication bias

**Fig. 4:**
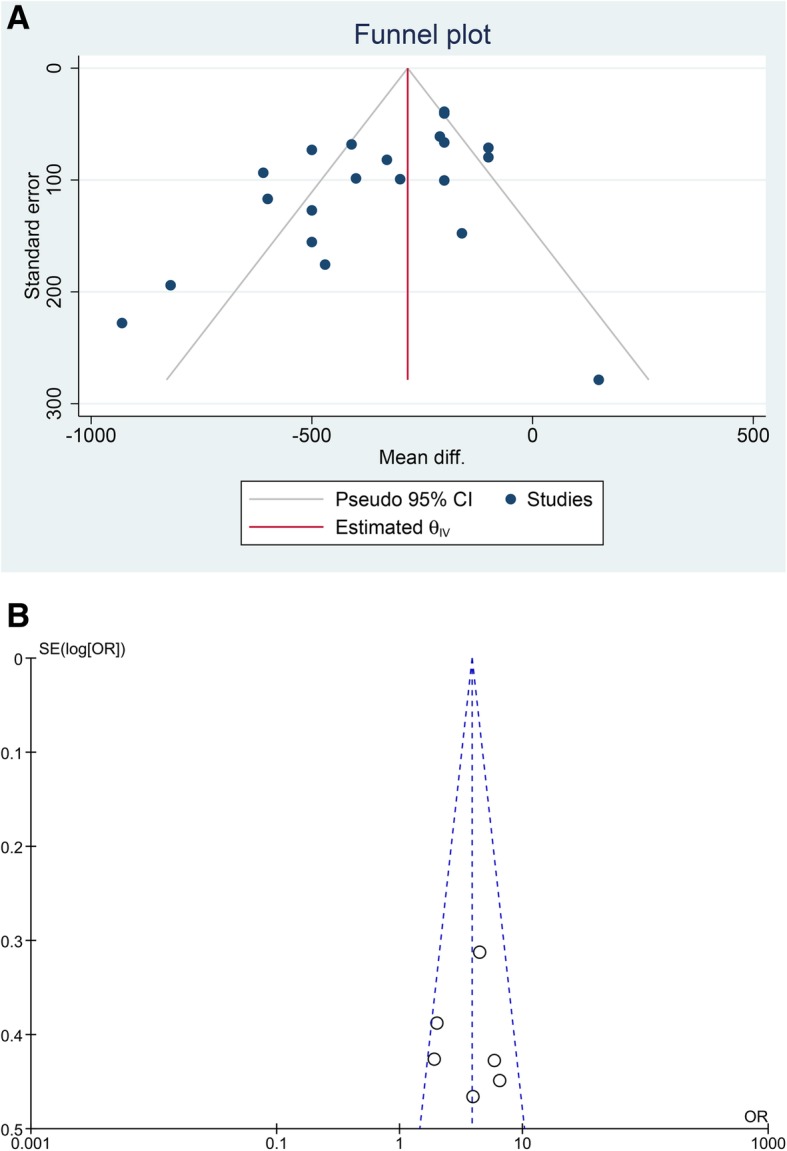
Publication bias. Funnel plot analysis showed asymmetrical shape for composite poor outcome and lymphocyte count (**a**), but symmetrical shape for lymphopenia and composite poor outcome (**b**)

Funnel plot analysis showed asymmetrical shape for lymphocyte count and composite poor outcome (Fig. 4). The funnel plot was symmetrical for lymphopenia and severe COVID-19. Regression-based Egger’s test showed statistically significant small-study effects (*p* = 0.018) for the lymphocyte and composite poor outcome. Trim-and-fill method did not impute any study. Regression-based Harbord’s test showed no evidence of small-study effects (*p* = 0.086) for lymphopenia and severe COVID-19 outcome.

## Discussion

This meta-analysis showed that lower lymphocyte count was associated with increased mortality, ARDS, need for ICU care, and severe COVID-19. The association seemed to be stronger in younger patients compared to older patients.

Although the definition of lymphopenia differed among studies, a subgroup analysis using ≤ 1100 cells/μL cut-off point has showed a consistent outcome in four studies [10–12, 19]. We set a cut-off point of ≤ 1100 μL because there were 4 studies using it as a cutoff point. There were only 2 studies for ≤ 1000 μL, and 1 study for < 1200μL and ≤1500 μL, respectively. This subgroup analysis  aimed to determine the magnitude of odds ratio at a specific cutoff point (not because of its superiority over the other cutoff points).

Based on the meta-regression result, subgroup analysis of age group by using 55 years old as the cutoff point was performed. By analyzing the bubble plot chart, the center of bubble plot is approximately 52 to 55 years old. Hence, we chose 55 as the cutoff point to ensure the number of studies is almost equal in the left side and the right side of the bubble plot. If the number of studies was too small, the pooled effect estimate will be less reliable. Interestingly, we found that the association between lymphopenia and severe COVID-19 was stronger in younger patients compared to older patients. This was a novel finding which, as far as we know, has not been discussed in previous literature. Although changes in the number and composition of lymphocytes are considered as hallmark of immunosenescence [30], it could not fully explain this association. One possible hypothesis is that the aging of the immune system could contribute to a relatively “non-reactive” immune state, thereby causing a relatively stable reduced lymphocyte count, while in younger populations, the highly active lymphocyte kinetics may be influenced by a wide range of insults and comorbidities, thus contributing to a relatively higher mean difference between younger populations.

This is further reflected by the sensitivity analysis which showed that upon removal of Wang et al. study, heterogeneity can be reduced to 0% for the ICU care outcome. This heterogeneity was attributed to the mean/median age; there were 3 studies for the ICU care outcome, Cao et al. (50.1 years old), Wang et al. (56 years old), and Huang et al. (49 years old). The difference between mean/median age of Cao et al. and Huang et al. was only 1.1 years old. This explains why the heterogeneity between Cao et al. and Huang et al. was low (0%).

Pre-existing cardiac disease has been shown to increase mortality in patients with COVID-19 [20]; in this meta-analysis, cardiac comorbidity was not found to affect the association between lymphocyte count difference and composite poor outcome. Angiotensin-converting enzyme (ACE) inhibitor and angiotensin-receptor blocker (ARB) have been hypothetically suggested to exacerbate COVID-19 due to increase in angiotensin II level [31]. These drugs are frequently used in patients with diabetes and hypertension, which was associated with poor outcome [32, 33]. Although we did not have data on hypertensive medications in the present study, meta-regression showed that hypertension and diabetes did not significantly affect the lymphocyte count difference between poor and good outcome.

Our understanding of the pathogenesis of lymphocyte reduction in COVID-19 might possibly be enlightened by studies of other similar beta-CoV infection, including severe acute respiratory syndrome (SARS)-CoV and Middle East respiratory syndrome (MERS)-CoV [34]. Peripheral T lymphocytes, both CD4+ and CD8+, are rapidly reduced in acute SARS-CoV infection hypothetically due to lymphocyte sequestration in specific target organs [35]. Although MERS-CoV and SARS-CoV are structurally similar, they bind to different receptors to facilitate entry. SARS-CoV attaches to angiotensin-converting enzyme 2 (ACE2) to enter the host cells, while MERS-CoV attaches to a different receptor, namely dipeptidyl peptidase 4 (DPP4) [36]. Although the mechanism of significant lymphocyte reduction in severe COVID-19 remains unclear, there are hypothesis other than lymphocyte infiltration and sequestration in the lungs, gastrointestinal tracts, and or lymphoid tissues: (1) lymphocytes express the ACE2 receptor and may be a direct target of SARS-CoV-2 infection [37], and (2) an increase of pro-inflammatory cytokines in COVID-19, especially IL-6, could induce further lymphocyte reduction [34].

### Implication for clinical practice

Lymphopenia can be used as a marker for poor prognosis in COVID-19 and in younger patients in particular. Lymphopenia defined as lymphocyte count ≤ 1100 cells/μL is associated with threefold risk of poor outcome.

### Limitation

The limitation of this systematic review and meta-analysis is the presence of publication bias. This is apparent in the lymphocyte count and composite poor outcome. Most of the articles included in the study were published at preprint server of which are not yet peer-reviewed. Data curation from preprint server is crucial due to the novel and emergent nature of COVID-19; most of the studies are not yet published in journals. Most of the studies were exclusively from China; thus the possibility of the same patients reported more than once is high and may represent inaccurate scientific records. The included studies were also mostly retrospective in design. We encourage further studies to create prognostic model that include lymphopenia.

## Conclusion

This meta-analysis showed that lymphopenia on admission was associated with poor outcome in patients with COVID-19.

## Supplementary information

**Additional file 1: Table S1.** Electronic search strategy.

**Additional file 2: Figure S1.** Subgroup analysis performed for lymphopenia.

## Data Availability

All data generated or analyzed during this study are included in this published article. Corresponding author (R.P) can be contacted for more information.

## Abbreviations

ACE: Angiotensin-converting enzyme
ACE2: Angiotensin-converting enzyme 2
ARB: Angiotensin receptor blocker
ARDS: Acute respiratory distress syndrome
COVID-19: Coronavirus disease 2019
DPP4: Dipeptidyl peptidase 4
ICU: Intensive care unit
MERS-CoV: Middle East respiratory syndrome coronavirus
MOF: Multi-organ failure
MD: Mean difference
OR: Odds ratio
SARI: Severe acute respiratory infection
SARS-Cov: Severe acute respiratory syndrome coronavirus
SD: Standard deviation
WHO: World Health Organization
