# Supplementary material for: Lymphopenia in severe coronavirus disease-2019 (COVID-19): systematic review and meta-analysis
Source: J Intensive Care. 2020 May 24;8:36. doi: 10.1186/s40560-020-00453-4 (PMC7245646; doi:10.1186/s40560-020-00453-4)
Supplement: Supplementary file 1 — Additional file 1: Table S1. Electronic search strategy. [file 40560_2020_453_MOESM1_ESM.docx]

**Table S1. Electronic search strategy**

| **Search Engine** | **Keywords** | **Records** |
| --- | --- | --- |
| PubMed | COVID-19[All Fields] OR "SARS-CoV-2"[All Fields] AND "Lymphocyte"[All Fields] | 13 |
| SCOPUS | "COVID-19" OR "SARS-CoV-2" AND "Lymphocyte" | 6 |
| EuroPMC | "COVID-19" OR "SARS-CoV-2" AND "Lymphocyte" | 57 |
| ProQuest | "COVID-19" OR "SARS-CoV-2" AND "Lymphocyte" | 62 |
| Cochrane Central | "COVID-19" OR "SARS-CoV-2" AND "Lymphocyte" | 2 |
| Google Scholar | "COVID-19" OR "SARS-CoV-2" AND "Lymphocyte" | 10* |

*10 records of potential articles were obtained from Google Scholar
