# Supplementary figures and images for: Lymphopenia in severe coronavirus disease-2019 (COVID-19): systematic review and meta-analysis
Source: J Intensive Care. 2020 May 24;8:36. doi: 10.1186/s40560-020-00453-4 (PMC7245646; doi:10.1186/s40560-020-00453-4)

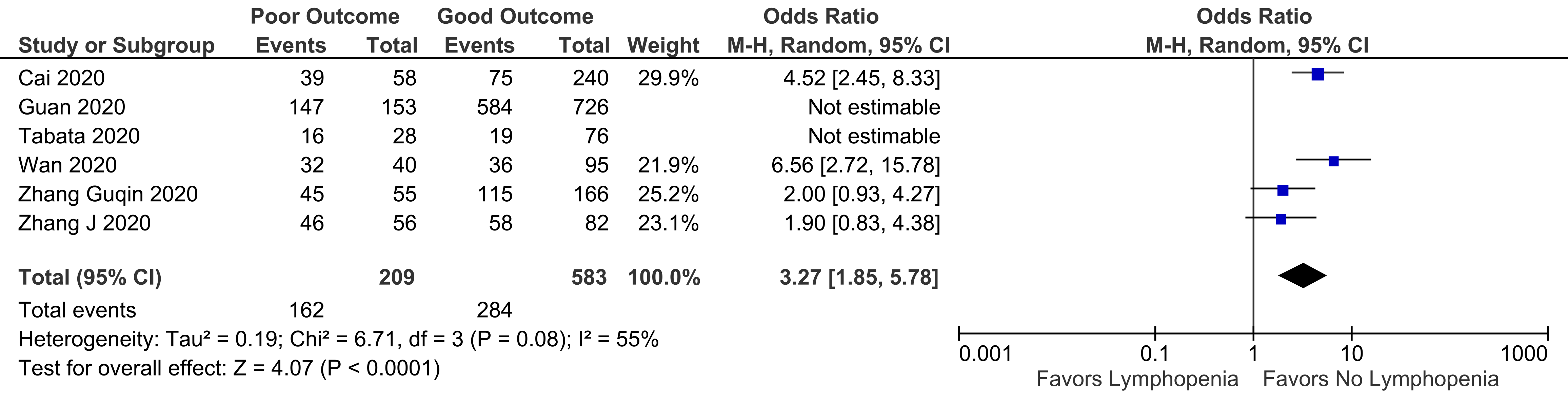

Supplement: Supplementary file 2 — Additional file 2: Figure S1. Subgroup analysis performed for lymphopenia. [file 40560_2020_453_MOESM2_ESM.tif]
